# Altered Gray Matter Volume and Functional Connectivity in Patients With Vestibular Migraine

**DOI:** 10.3389/fnins.2021.683802

**Published:** 2021-07-08

**Authors:** Xia Zhe, Xiaoling Zhang, Li Chen, Li Zhang, Min Tang, Dongsheng Zhang, Longchao Li, Xiaoyan Lei, Chenwang Jin

**Affiliations:** ^1^Department of Radiology, The First Affiliated Hospital of Xi’an Jiaotong University, Xi’an, China; ^2^Department of MRI, Shaanxi Provincial People’s Hospital, Xi’an, China; ^3^Department of Neurology, Shaanxi Provincial People’s Hospital, Xi’an, China

**Keywords:** vestibular migraine, vertigo, gray matter volume, voxel-based morphometry, resting-state functional connectivity

## Abstract

**Subjects:**

Vestibular migraine (VM) is the most common neurological cause of vertigo in adults. Previous neuroimaging studies have reported structural alterations in areas associated with pain and vestibular processing. However, it is unclear whether altered resting-state functional connectivity (FC) exists in brain regions with structural abnormalities in patients with VM.

**Methods:**

Resting-state functional magnetic resonance imaging (MRI) and three-dimensional T1-weighed MRI were performed in 30 patients with VM and 30 healthy controls (HCs). Patients underwent an evaluation of migraine and dizziness severity. FC and voxel-based morphometry (VBM) were performed using DPABI 4.3 and CAT12, respectively. The association between changes in gray matter (GM) volume or FC and clinical parameters was also analyzed.

**Results:**

Compared with HCs, patients with VM demonstrated a reduced GM volume in the bilateral parietoinsular vestibular cortex (PIVC), right middle frontal gyrus, and precuneus. The GM volume of the left PIVC was negatively associated with Dizziness Handicap Inventory score in patients with VM. Taking this region as a seed region, we further observed increased FC between the left primary somatosensory cortex (S1)/inferior parietal lobule (IPL) and the left PIVC in patients with VM.

**Conclusion:**

FC between regions with a decline in GM volume (the PIVC and S1/IPL) is altered in patients with VM, suggesting that abnormalities in vestibular cortical network could be useful for understanding the underlying mechanisms of VM.

## Introduction

Vestibular migraine (VM) is the most common neurological cause of vertigo, with a reported prevalence of between 1 and 2.7% in the adult population (Neuhauser et al., 2006; Formeister et al., 2018). Recently, the Bárány Society and the International Headache Society published a consensus on diagnostic criteria for VM (Lempert et al., 2012). This condition has been added to the third edition of the International Classification of Headache Disorders (Headache Classification Committee of the International Headache Society, 2013). At present, the exact pathophysiology of VM is incompletely understood. Fortunately, development of neuroimaging techniques has provided opportunities to deepen our knowledge of the mechanisms that underpin VM.

Neuroimaging methods have been adopted to study structural and functional brain alterations in patients with VM, even if only a few studies using VBM have revealed abnormal gray matter (GM) volume in patients with VM. However, VBM results are conflicting in patients with VM. Obermann et al. found that loss of GM volume in cortical and subcortical regions is mainly observed in the temporal lobe (superior, inferior, and middle temporal gyri) and the middle cingulate, dorsolateral prefrontal, insular, parietal, and occipital cortices in patients with VM compared with controls (Obermann et al., 2014). Messina and Wang et al. identified an increase in GM volume in the frontal lobe, as well as occipital and angular regions, in patients with VM compared with controls (Shin et al., 2014; Wang et al., 2019). Previous studies suggest that recurring migraine and vertigo attacks over time may lead to selective damage to several brain regions involved in pain and visual and vestibular processing. Furthermore, VM may have cumulative effects on brain structure, because some alterations may be associated with a longer disease duration and increased migraine frequency. These divergencies in GM volume abnormalities could be influenced by certain factors, such as the demographic characteristics of subjects, including white matter hyperintensity, sex, illness duration, and data acquisition and processing. Thus, the study enrolled patients with VM without white matter hyperintensity and used voxel-wise whole-brain methods to avoid selection bias when using traditional labor-intensive regions of interest.

Functional neuroimaging methods have also been used to study functional abnormalities in the brain in patients with VM. Positron emission tomography studies revealed an increase in metabolism in temporo-parieto-insular areas and bilateral thalami during VM attacks (Shin et al., 2014), which indicate activation of the vestibulo-thalamo-cortical pathway. Moreover, a recent functional magnetic resonance imaging (fMRI) study demonstrated activation of brain areas related to integration of visual and vestibular cues in two patients with VM during a visual stimulation procedure in a vertigo-free period (Teggi et al., 2016). Recently, Russo et al. used whole-brain blood oxygen level-dependent (BOLD) fMRI during caloric irrigation. They showed significantly increased thalamic activation in 12 patients with VM in a vertigo-free period compared with patients with migraine and healthy individuals. In addition, the magnitude of thalamic activation positively correlated with the frequency of migraine attacks in patients with VM. Functional imaging demonstrated that thalamic dysfunction is involved in central vestibular processing (Russo et al., 2014). Although previous studies have demonstrated structural and functional alterations in the brain in patients with VM, these studies used a single-mode MRI method. It is still unknown whether functional changes are related to structural changes. Moreover, to our knowledge, no prior studies have assessed resting-state changes in FC in brain regions with structural abnormalities.

In this study, we aimed to employ multi-modal imaging approaches by combining VBM and resting-state FC analyses to identify changes in GM volume and identify abnormal FC in patients with VM during the interictal period. In light of a previous study (Zhe et al., 2020), we hypothesized that patients with VM would exhibit abnormalities in FC in brain regions with structural alterations compared with healthy controls (HCs). Clinical information was used to assess the relationship between neuroimaging findings and VM symptoms. This combination of structural and functional methods may enhance our understanding of the neural mechanisms of VM.

## Materials and Methods

### Subjects

According to the International Classification of Headache Disorders, 30 patients with VM (24 without aura and six with aura) were recruited from the vertigo and dizziness outpatient service center of Shaanxi Provincial People’s Hospital in China between January 2016 and October 2020 (Headache Classification Committee of the International Headache Society, 2013). All patients with VM were right-handed. No neurological, psychiatric, audio-vestibular, or systemic disorders were reported.

To avoid any possible pharmacological interference with BOLD signal changes, patients with VM did not take medications for at least 3 days before the fMRI scan. Moreover, MRI scans were performed on days 3–7 after a VM attack, and all patients were required to be attack-free during the experiment. All patients underwent a routine neurological and neuro-otological examination, as well as MRI scanning, which were performed on the same day. No peripheral vestibular dysfunction was found on videonystagmography recordings. All patients underwent examination using the Visual Analog Scale (0, no pain; 10, worst possible pain); the Migraine Disability Assessment Scale; the Headache Impact Test-6 and the Dizziness Handicap Inventory (DHI) using a face-to-face interview with a standardized questionnaire (Sauro et al., 2010; Hawker et al., 2011; Balci et al., 2018). Twelve patients with VM were treated with migraine-preventive medication and non-steroidal analgesics. The majority of patients (*n* = 18) did not take any regular medication.

Thirty HCs who were matched for age, sex, and education were from the community. The exclusion criteria were as follows: left-handedness; migraine; chronic pain; previous vestibular neuritis; Meniere’s disease; secondary somatoform vertigo; drug abuse; neurological, mental, or systemic disorders; ischemic or hemorrhagic stroke; and severe head trauma. All subjects had no structural abnormalities or visible T2-weighted hyperintensities in deep white matter on MRI.

This study was approved by the Ethics Committee of Shaanxi Provincial People’s Hospital. All participants provided written informed consent before participation.

### Imaging Data Acquisition

Experimental data were acquired using a 3.0-T Philips Ingenia scanner with a 16-channel phased-array head coil. A high-resolution three-dimensional magnetization-prepared rapid-acquisition gradient echo T1-weighted sequence covering the whole brain (332 sagittal slices) was used. The acquisition parameters were as follows: repetition time = 1900 ms; echo time = 2.26 ms; inversion time = 900 ms; flip angle = 9°; matrix = 256 × 256; field of view = 220 × 220 mm; slice thickness = 1.00 mm; no interslice gap. Resting-state functional BOLD images were scanned using gradient echo planar imaging with the following parameters: repetition time = 2000 s; echo time = 30 ms; slices = 34; slice thickness = 4 mm; slice gap = 0 mm; field of view = 230 × 230 mm; matrix = 128 × 128; flip angle = 90°; and 200 volumes. As for the resting-state scan, all subjects were asked to keep their eyes closed, to keep their minds calm, and to stay awake throughout the scan. After the scan, subjects were asked whether or not they remained awake during the whole procedure.

### Image Processing

Structural scans were processed using CAT12^1^ for SPM12 in MATLAB R2014b (MathWorks, Inc.). CAT12 is one of the most important neuroimaging analysis approaches used to examine structural alterations in regional GM volume (Besteher et al., 2017). Moreover, CAT12 can avoid operational bias when selecting brain regions and performing automated whole-brain measurements. This toolbox includes bias-field and noise removal; skull stripping; and GM, white matter, and cerebrospinal fluid segmentation. Afterward, all GM images were normalized to the standard Montreal Neurological Institute template using diffeomorphic anatomical registration and exponential Lie algebra (DARTEL) to a 1.5-mm isotropic adult template provided by the CAT12 toolbox (Ashburner, 2007). The resulting images were checked for homogeneity. As all images had high correlation values (>0.85), no images were discarded. Finally, GM images were smoothened using an 8-mm full width at half maximum Gaussian kernel.

The original data of resting-state BOLD images were analyzed using a public toolbox named DPABI (for Data Processing & Analysis of Brain Imaging,^2^). To ensure stability of the BOLD signal, we removed the first 10 volumes. Scans were slice–time-corrected and realigned to the first scan in the experiment for correction of head motion. Any subjects whose mean frame-wise displacement (FD_Jenkinson) >0.2 mm were excluded (Yan et al., 2016). Data were normalized to echo planar imaging standard templates using DPABI and resampled to 3.0 mm × 3.0 mm × 3.0 mm. Images were smoothened using a Gaussian kernel with a full width at half maximum of 6 mm. Covariates, such as linear drift and cerebrospinal fluid, were removed, and the data were filtered to 0.01–0.1 Hz.

Functional connectivity was assessed using a method based on a seeding voxel correlation approach (Lui et al., 2009; Yuan et al., 2010; Jin et al., 2013). Regions with altered GM volume between VM patients and HCs were defined as seeding areas in the FC analysis. The reference time series for each seeding area was obtained by averaging the fMRI time series for all voxels within each of the regions with anatomical deficits.

### Statistical Analysis

#### Demographic and Clinical Data

Group differences in demographic variables were examined using independent t-tests and analysis of covariance in SPSS 22.0. A *P*-value of <0.05 was considered statistically significant (Table 1).

**TABLE 1:** Demographic and clinical characteristics of patients.

Characteristics	VM (*n* = 30) Mean ± SD	HC (*n* = 30) Mean ± SD	*P-*value
Sex (female/male)	27/3	26/4	0.69
Age (years)	39.67 ± 11.10	37.67 ± 12.14	0.51
Education (years)	13.63 ± 3.46	14.77 ± 2.00	0.13
Disease duration (months)	100.70 ± 86.09		
Headache frequency (number)	6.33 ± 4.89		
VAS	5.23 ± 2.33		
MIDAS	52.17 ± 48.01		
HIT-6	55.93 ± 12.74		
DHI	47.93 ± 15.07		

*VM, vestibular migraine; HC, healthy control; VAS, Visual Analog Scale (0 = no pain, 10 = worst possible pain); MIDAS, Migraine Disability Assessment Scale; HIT-6, Headache Impact Test-6; DHI, Dizziness Handicap Inventory.*

#### VBM Analysis

Gray matter volume was compared between patients with VM and HCs using two-sample t-tests in SPM 12 with age, sex, and total intracranial volume as covariates. Family-wise error (FWE) correction was performed for multiple comparison correction. A *P-*value of <0.05 was considered statistically significant. Thereafter, we extracted the average values of regions with decreased GM volume and performed a partial correlation analysis with all parameters, including disease duration, attack frequency, Visual Analog Scale score, Migraine Disability Assessment Scale score, Headache Impact Test-6 score, and DHI score. Age was controlled as a covariate. The significance threshold was set at *P* < 0.05. To evaluate the effects of clinical variables on the whole brain GM volume, we also performed correlation analysis based on the whole brain voxel level using SPM12. Age was controlled as a covariate. FWE corrected for multiple comparisons at a threshold of *P* < 0.05.

#### Functional Analysis

To calculate the difference in FC, the averaged time series of each region of interest (ROI) and the time series of every other voxel in the whole brain mask were extracted to calculate the correlation coefficients (r), after which Fisher’s *r*-to-*z* transformation was performed to improve the normality of the resulting r values and create the FC map for each participant. A comparison of FC between groups was performed using a two-sample *t*-test within the DPABI, while age, sex, and FD as covariates. Multiple comparisons correction was performed using a Gaussian random field at *P* < 0.05 (voxel *P* < 0.001, cluster size >33).

Finally, we extracted the average *Z*-values for each region with significant differences and performed a partial correlation analysis with patients’ clinical parameters using SPSS 22.0. Age, sex, and FD were controlled as covariates. The significance threshold was set at *P* < 0.05.

## Results

### Clinical Data

The clinical and demographic characteristics of the VM and HC groups are summarized in Table 1. No significant differences were found in age, sex, or years of education between patients with VM and HCs (*P* > 0.05; Table 1).

### VBM Results

**FIGURE 1:**
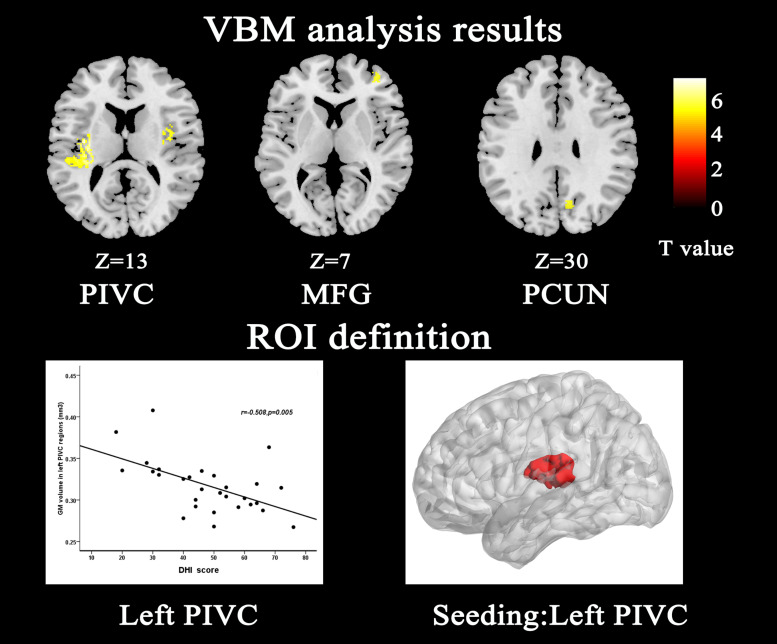
Structural analysis results of patients with vestibular migraine compared with healthy controls (*P* < 0.05, FWE corrected, with *k* > 100 voxels). Gray matter volume of the left parieto-insular vestibular cortex (PIVC) negatively correlated with Dizziness Handicap Inventory score; therefore, we chose the left PIVC as the seeding area for the functional connectivity analysis. The seeding area is indicated by red color. MFG middle frontal gyrus, PCUN Precuneus.

Compared with HCs, patients with VM showed significantly decreased GM volume in the left posterior insula, parietal operculum, superior temporal gyrus (parieto-insular vestibular cortex [PIVC]), right middle frontal gyrus, right posterior insular/parietal operculum region, and precuneus (Table 2 and Figure 1). No significant increase in GM volume was detected. Only GM volume in the left PIVC showed a significant negative correlation with DHI score in patients with VM (*r* = −0.508; *P* = 0.005; Figure 1). Based on whole brain voxel-wise correlation analysis, there was no correlation between GM volume and clinical variables.

**TABLE 2:** Decreased gray matter volume in various brain regions in patients with vestibular migraine.

	Brain regions	Peak MNI			Cluster voxels	*T*	*Z*	*P*
R	Middle frontal gyrus	38	50	5	152	5.69	5.05	0.000
	Posterior insula/parietal operculum	38	−6	14	128	5.60	4.99	0.000
	Precuneus	12	−68	26	132	5.90	5.20	0.000
L	Posterior insula	−36	−11	14	1365	7.15	6.03	0.000
	Parietal operculum	−39	−29	16	301	7.15	6.03	0.000
	Superior temporal gyrus	−43	−29	11	455	7.15	6.03	0.000

*MNI, Montreal Neurological Institute; R, right; L, Left.*

### FC Results

**FIGURE 2:**
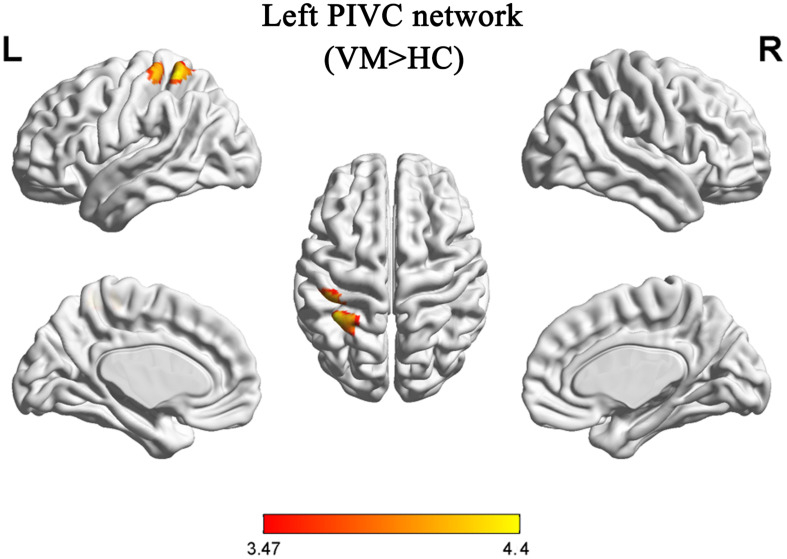
Functional analysis results of the left parieto-insular vestibular cortex (PIVC) during the resting state in patients with vestibular migraine compared with healthy controls (*P* < 0.05, Gaussian random field-corrected, with *k* > 33 voxels).

Compared with HCs, patients with VM showed increased FC between the primary somatosensory cortex (S1)/inferior parietal lobule (IPL) and the left PIVC (Table 3 and Figure 2). No regions showed a significant decrease in FC with the left PIVC in patients with VM.

**TABLE 3:** Brain regions with increased functional connectivity in patients with vestibular migraine compared with healthy controls.

Seed ROI	Brain region	Peak MNI			Cluster voxels	*T*
L posterior insula/parietal regions/superior temporal gyrus	L primary somatosensory cortex/inferior parietal lobule	−42	−42	66	56	4.40

*ROI, region of interest; MNI, Montreal Neurological Institute; L, left.*

The correlation analysis failed to identify any significant correlation between changes in FC and clinical characteristics in patients with VM.

## Discussion

In the present study, we used VBM and FC analyses to evaluate functional and structural changes in GM patterns in patients with VM. Compared with HCs, reduced GM volume was observed in the bilateral PIVC, right middle frontal gyrus, and precuneus in the VM group. In addition, there was an increase in FC between the left part of PIVC where GM volume was reduced, and the left part S1/IPL. Our present data demonstrate changes in FC in resting-state networks of brain regions with structural alterations. These findings suggest that abnormalities in vestibular cortical network may be involved in associated with the pathophysiology of VM.

To date, few studies have identified structural and functional abnormalities in the brain in patients with VM (Obermann et al., 2014; Russo et al., 2014; Messina et al., 2017; Wang et al., 2019; Zhe et al., 2020). However, previous researchers only used single-mode MRI methods, and whether changes in FC exist in brain regions with structural alterations during the resting state in patients with VM remains unclear. In the current study, an attempt was made to identify an association between GM volume and alterations in FC in patients with VM by combining VBM and FC to elucidate the mechanism of central pain processing in VM.

The main finding in the present study was that there was a significant loss of GM volume in the PIVC in patients with VM relative to HCs, which is in line with previous studies (Obermann et al., 2014; Zhe et al., 2020). This brain region is recognized as the core region of the vestibular cortex in humans. The human vestibular cortex is located in the posterior parietal operculum/retro-insular region and extends into the posterior insular lobe (Eickhoff et al., 2006; zu Eulenburg et al., 2012). There is general agreement that the region described as the PIVC in humans is critical to vestibular processing (Lopez and Blanke, 2011; Lopez et al., 2012; zu Eulenburg et al., 2012; Dieterich and Brandt, 2015, 2018). VBM studies identified a reduction in GM volume in the posterior insula, cingulate cortex, and inferior temporal gyrus in patients with VM, suggesting that these areas are involved in cortical processing of vestibular and nociceptive information (Obermann et al., 2014). Furthermore, impaired GM volume in the PIVC was negatively correlated with DHI score in the present study. The correlation results demonstrated that recurring long-term headache and vertigo attacks have a cumulative effect on GM volume. Our results indicated that abnormal GM volume might contribute to dysfunction in information processing, which supports the hypothesis that impaired GM volume is associated with the pathophysiology of VM.

As GM volume of the PIVC was reduced in patients with VM and it exhibited a negative correlation with the DHI score, we chose this region as our ROI to investigate resting-state FC changes in patients with VM. Patients with VM showed abnormal FC between the PIVC and the S1/IPL relative to HCs in the present study. Similar to our results, a recent fMRI study showed abnormal FC between the S1 and the insular cortex in individuals who suffer migraine (Zhang et al., 2017). Results from human neuroimaging studies show that the parietal lobe and somatosensory and insular cortices belong to the vestibular network (Ventre-Dominey, 2014; Dieterich and Brandt, 2015; Smith et al., 2017). Previous studies have shown that the insula is related to triggering of the pain matrix network and to the subjective pain experience (Starr et al., 2009). Increasing evidence indicates that the insula may be a cortical hub involved in processing complex sensory and emotional information during migraine (Menon and Uddin, 2010). The IPL is involved in spatial discrimination and attention to pain (Maihöfner et al., 2006, 2010; Burgmer et al., 2011). Evidence for a role of the IPL in the neuropathology of VM has been reported. Patients with VM exhibit increased activation of the IPL compared with controls (Teggi et al., 2016). We speculated that dysfunction in FC between the IPL and the PIVC in VM patients during a vertigo-free period might contribute to spatial attention and reorientation. Some reports have shown that the S1 could provide information about the intensity, localization, and temporal attributes of noxious stimuli. Reports also suggest S1 involvement in pain perception (D R Kenshalo and Isensee, 1983; Ogino et al., 2005). Other studies propose that the S1 is chiefly involved in discriminating sensory features of pain, and it also plays an active role in the afferent filtering module, as well as the intensity and spatial discriminative pain pathways of the pain-processing system (Hofbauer et al., 2001; Oshiro et al., 2007, 2009). Activation of the S1 has been reported in approximately 75% of human imaging studies on pain (Apkarian et al., 2005). Burstein et al. (2015) and Goadsby et al. (2017) demonstrated that the S1 and the secondary somatosensory cortex, primary motor cortex, secondary motor cortex, and insular cortex are in the trigeminovascular pathway and are implicated in the ascending trigemino-thalamo-cortical nociceptive pathway. Therefore, our results observed GM volume abnormalities in the PIVC and found that dysfunction in FC between the S1 and the PIVC may disrupt the pathway that discriminates sensory features of pain, and the flow of information between the vestibular system and the cortex. Therefore, it is possible to speculate that the GM volume reductions of the PIVC led to a systems-level alteration in FC. At present, due to the causal relationship between GM volume and FC is unknown, more study is need to reveal this puzzling question. The approach in our study combines the GM structural and FC alterations in patients with VM, which may help to understand underlying the mechanisms of VM.

As part of the prefrontal cortex, the middle frontal gyrus is thought to be linked to pain perception and management. Previous studies have described human vestibular areas in detail, including the middle frontal gyrus and other brain regions (Bottini et al., 2001; Dieterich et al., 2003; Indovina et al., 2005; Stephan et al., 2005). In concordance with previous research (Obermann et al., 2014; Zhe et al., 2020), we found decreased GM volume in patients with VM in the middle frontal gyrus, which is thought to be part of the pain processing network (Koechlin and Hyafil, 2007). The frequency of migraine and vertigo attacks has a significant impact on GM volume in the middle frontal gyrus (Obermann et al., 2014). In contrast, according to a recent VBM study, patients with VM exhibit increased GM volume in the medial superior frontal gyrus and angular gyrus compared with HCs (Wang et al., 2019). These inconsistent results may have emerged from demographic inconsistencies, such as differences in the frequency of attacks, disease duration, and medication, in some patients with VM with GM structural changes. However, meta-analyses deemed that the prefrontal cortex is the most crucial brain region associated with structural changes in patients with migraine (Hu et al., 2015; Jia and Yu, 2017). GM volume in the middle frontal gyrus changed in patients with VM in the present study, suggesting that disruption to the sensory network causes abnormal modulation of pain perception.

Our research also obtained interesting findings in the precuneus. The VBM analysis found decreased GM volume in the precuneus in the VM group compared with the HC group. We identified a region that had not been reported previously in patients with VM compared with HCs. The precuneus is involved in information transfer and multi-modal integration, which might be crucial for processing of spontaneous thoughts for internal awareness (Tomasi and Volkow, 2011). The precuneus is a pivotal node of the default mode network, which is particularly sensitive to cognitive states in self-referential processing (Raichle et al., 2001). Studies have shown that FC in the default mode network changes in various pain conditions (Baliki et al., 2014). The current study suggests that chronic pain might disrupt default mode network activity. Although our data show loss of GM volume in the precuneus in patients with VM, the function of the precuneus is complicated. Specifically, dysfunction in the precuneus is not only observed in VM, but also in many other pain disorders, such as migraine (Yang et al., 2019), chronic pain (Malfliet et al., 2019), diabetic neuropathic pain (Cauda et al., 2009), and fibromyalgia (Napadow et al., 2010). These studies indicate that structural damage in the precuneus is not specific to VM.

To our knowledge, this is the first study to combine VBM with resting-state FC analysis to explore changes in FC in brain regions with structural alterations in patients with VM. However, our study has some limitations. First, the sample size was relatively small; nevertheless, all subjects were recruited for long-term follow-up observations. Second, the whole brain voxel-wise correlation analysis between GM volume and clinical variables produced no significant results. Thus, further studies should pay more attention to the effects of clinical symptom on the whole brain structure.Third, a comparative study of migraine subtypes, such as migraine without aura and migraine with aura, was not performed, because the majority of patients enrolled had migraine without aura. A subtype comparative study will be performed in the future. Fourth, due to the fact that the sample size was relatively small, this study was not carried out the mediation analysis to clarify relationship among migraine and vertigo-related clinical symptoms, GM volume, and FC. Future studies should utilize the method to clarify relationship between the three when we recruit a larger sample size. Furthmore, we cannot exclude that the use of preventive medication might have influenced structural and functional. Finally, because this was a cross-sectional study, we could not determine the causal relationship between disease and structural/functional brain alterations. Longitudinal studies may help to clarify this shortcoming.

## Conclusion

In summary, using a multi-modal neuroimaging approach, we detected structural and functional changes in the brain in patients with VM. We found changes in GM volume in the PIVC accompanied by changes in FC. These findings suggest that abnormalities in vestibular cortical network might be useful for understanding the underlying mechanisms of VM.

## Data Availability Statement

The original contributions presented in the study are included in the article/Supplementary Material, further inquiries can be directed to the corresponding author/s.

## Ethics Statement

The studies involving human participants were reviewed and approved by the Ethics Committee of Shaanxi Provincial People’s Hospital. The patients/participants provided their written informed consent to participate in this study.

## Author Contributions

XZhe drafted the manuscript, designed the experiment, and performed the statistical analysis. LC undertook the clinical parameters assessments. LZ, MT, LL, and DZ collected the data. XL and XZha provided technical support. CJ performed the study’s supervision or coordination. All authors read and approved the final manuscript.

## Conflict of Interest

The authors declare that the research was conducted in the absence of any commercial or financial relationships that could be construed as a potential conflict of interest.

## Footnotes

1 http://dbm.neuro.uni-jena.de/cat12/

2 http://rfmri.org/dpabi

## References

[B1] Apkarian A. V. Bushnell M. C. Treede R. D. Zubieta J. K. (2005). Human brain mechanisms of pain perception and regulation in health and disease. *Eur. J. Pain* 9 463–484. 10.1016/j.ejpain.2004.11.001 15979027

[B2] Ashburner J. (2007). A fast diffeomorphic image registration algorithm. *Neuroimage* 38 95–113. 10.1016/j.neuroimage.2007.07.007 17761438

[B3] Balci B. Senyuva N. Akdal G. (2018). Definition of balance and cognition related to disability levels in vestibular migraine patients. *Noro Psikiyatr Ars.* 55 9–14. 30042635 10.29399/npa.12617 PMC6045802

[B4] Baliki M. N. Mansour A. R. Baria A. T. Apkarian A. V. (2014). Functional reorganization of the default mode network across chronic pain conditions. *PLoS One* 9:e106133. 10.1371/journal.pone.0106133 25180885 PMC4152156

[B5] Besteher B. Gaser C. Langbein K. Dietzek M. Sauer H. Nenadic I. (2017). Effects of subclinical depression, anxiety and somatization on brain structure in healthy subjects. *J. Affect. Disord.* 215 111–117. 10.1016/j.jad.2017.03.039 28319687

[B6] Bottini G. Karnath H. O. Vallar G. Sterzi R. Frith C. D. Frackowiak R. S. (2001). Cerebral representations for egocentric space_ Functional-anatomical evidence from caloric vestibular stimulation and neck vibration. *Brain* 124 1182–1196. 10.1093/brain/124.6.1182 11353734

[B7] Burgmer M. Petzke F. Giesecke T. Gaubitz M. Heuft G. Pfleiderer B. (2011). Cerebral activation and catastrophizing during pain anticipation in patients with fibromyalgia. *Psychosom. Med.* 73 751–759. 10.1097/psy.0b013e318236588a 22048836

[B8] Burstein R. Noseda R. Borsook D. (2015). Migraine: multiple processes, complex pathophysiology. *J. Neurosci.* 35 6619–6629. 10.1523/jneurosci.0373-15.2015 25926442 PMC4412887

[B9] Cauda F. Sacco K. Duca S. Cocito D. D’agata F. Geminiani G. C. (2009). Altered resting state in diabetic neuropathic pain. *PLoS One* 4:e4542. 10.1371/journal.pone.0004542 19229326 PMC2638013

[B10] Dieterich M. Bense S. Lutz S. Drzezga A. Stephan T. Bartenstein P. (2003). Dominance for vestibular cortical function in the non-dominant hemisphere. *Cereb. Cortex* 13 994–1007. 10.1093/cercor/13.9.994 12902399

[B11] Dieterich M. Brandt T. (2015). The bilateral central vestibular system: its pathways, functions, and disorders. *Ann. N. Y. Acad. Sci.* 1343 10–26. 10.1111/nyas.12585 25581203

[B12] Dieterich M. Brandt T. (2018). The parietal lobe and the vestibular system. *Handb. Clin. Neurol.* 151 119–140. 10.1016/b978-0-444-63622-5.00006-1 29519455

[B13] Eickhoff S. B. Heim S. Zilles K. Amunts K. (2006). Testing anatomically specified hypotheses in functional imaging using cytoarchitectonic maps. *Neuroimage* 32 570–582. 10.1016/j.neuroimage.2006.04.204 16781166

[B14] Formeister E. J. Rizk H. G. Kohn M. A. Sharon J. D. (2018). The epidemiology of vestibular migraine: a population-based survey study. *Otol. Neurotol.* 39 1037–1044. 10.1097/mao.0000000000001900 30020261

[B15] Goadsby P. J. Holland P. R. Martins-Oliveira M. Hoffmann J. Schankin C. Akerman S. (2017). Pathophysiology of migraine: a disorder of sensory processing. *Physiol. Rev.* 97 553–622. 10.1152/physrev.00034.2015 28179394 PMC5539409

[B16] Hawker G. A. Mian S. Kendzerska T. French M. (2011). Measures of adult pain: Visual Analog Scale for Pain (VAS Pain), Numeric Rating Scale for Pain (NRS Pain), McGill Pain Questionnaire (MPQ), Short-Form McGill Pain Questionnaire (SF-MPQ), Chronic Pain Grade Scale (CPGS), Short Form-36 Bodily Pain Scale (SF-36 BPS), and Measure of Intermittent and Constant Osteoarthritis Pain (ICOAP). *Arthritis Care. Res. (Hoboken)* 63(Suppl. 11) S240–S252. 22588748 10.1002/acr.20543

[B17] Headache Classification Committee of the International Headache Society (2013). The international classification of headache disorders, 3rd edition (beta version). *Cephalalgia* 33 629–808. 10.1177/0333102413485658 23771276

[B18] Hofbauer R. K. Rainville R. Duncan G. H. (2001). Cortical representation of the sensory dimension of pain. *J. Neurophysiol.* 86 402–411. 10.1152/jn.2001.86.1.402 11431520

[B19] Hu W. Guo J. Chen N. Guo J. He L. (2015). A meta-analysis of voxel-based morphometric studies on migraine. *Int. J. Clin. Exp. Med.* 8 4311–4319. 26064347 PMC4443181

[B20] Indovina I. Maffei V. Bosco G. Zago M. Macaluso E. Lacquaniti F. (2005). Representation of visual gravitational motion in the human vestibular cortex. *Science* 308 416–419. 10.1126/science.1107961 15831760

[B21] Jia Z. Yu S. (2017). Grey matter alterations in migraine: a systematic review and meta-analysis. *Neuroimage Clin.* 14 130–140. 10.1016/j.nicl.2017.01.019 28180071 PMC5279908

[B22] Jin C. Yuan K. Zhao L. Zhao L. Yu D. Von Deneen K. M. (2013). Structural and functional abnormalities in migraine patients without aura. *NMR Biomed.* 26 58–64. 10.1002/nbm.2819 22674568

[B23] Kenshalo D. R. Isensee O. (1983). Responses of primate SI cortical neurons to noxious stimuli. *J. Neurophysiol.* 50 1479–1496. 10.1152/jn.1983.50.6.1479 6663338

[B24] Koechlin E. Hyafil A. (2007). Anterior prefrontal function and the limits of human decision-making. *Science* 318 594–598. 10.1126/science.1142995 17962551

[B25] Lempert T. Olesen J. Furman J. Waterston J. Seemungal B. Carey J. (2012). Vestibular migraine: diagnostic criteria. *J. Vestib. Res.* 22 167–172. 10.3233/ves-2012-0453 23142830

[B26] Lopez C. Blanke O. (2011). The thalamocortical vestibular system in animals and humans. *Brain Res. Rev.* 67 119–146. 10.1016/j.brainresrev.2010.12.002 21223979

[B27] Lopez C. Blanke O. Mast F. W. (2012). The human vestibular cortex revealed by coordinate-based activation likelihood estimation meta-analysis. *Neuroscience* 212 159–179. 10.1016/j.neuroscience.2012.03.028 22516007

[B28] Lui S. Deng W. Huang X. Jiang L. Ma X. Chen H. (2009). Association of cerebral deficits with clinical symptoms in antipsychotic-naive first-episode schizophrenia_ an optimized voxel-based morphometry and resting state functional connectivity study. *Am. J. Psychiatry* 166 196–205. 10.1176/appi.ajp.2008.08020183 18981063

[B29] Maihöfner C. Handwerker H. O. Birklein F. (2006). Functional imaging of allodynia in complex regional pain syndrome. *Neurology* 66 711–717. 10.1212/01.wnl.0000200961.49114.39 16534108

[B30] Maihöfner C. Jesberger F. Seifert F. Kaltenhäuser M. (2010). Cortical processing of mechanical hyperalgesia: a MEG study. *Eur. J. Pain* 14 64–70. 10.1016/j.ejpain.2009.02.007 19346142

[B31] Malfliet A. De Pauw R. Kregel J. Coppieters I. Meeus M. Roussel N. (2019). Gender differences in the association of brain gray matter and pain-related psychosocial characteristics. *Pain Physician* 22 E191–E203. 31151342

[B32] Menon V. Uddin L. Q. (2010). Saliency, switching, attention and control: a network model of insula function. *Brain Struct. Funct.* 214 655–667. 10.1007/s00429-010-0262-0 20512370 PMC2899886

[B33] Messina R. Rocca M. A. Colombo B. Teggi R. Falini A. Comi G. (2017). Structural brain abnormalities in patients with vestibular migraine. *J. Neurol.* 264 295–303. 10.1007/s00415-016-8349-z 27888414

[B34] Napadow V. Lacount L. Park K. As-Sanie S. Clauw D. J. Harris R. E. (2010). Intrinsic brain connectivity in fibromyalgia is associated with chronic pain intensity. *Arthritis Rheum.* 62 2545–2555. 10.1002/art.27497 20506181 PMC2921024

[B35] Neuhauser H. K. Radtke A. Von Brevern M. Feldmann M. Lezius F. Ziese T. (2006). Migrainous vertigo_ prevalence and impact on quality of life. *Neurology* 67 1028–1033. 10.1212/01.wnl.0000237539.09942.06 17000973

[B36] Obermann M. Wurthmann S. Steinberg B. S. Theysohn N. Diener H. C. Naegel S. (2014). Central vestibular system modulation in vestibular migraine. *Cephalalgia* 34 1053–1061. 10.1177/0333102414527650 24662323

[B37] Ogino Y. Nemoto H. Goto F. (2005). Somatotopy in human primary somatosensory cortex in pain system. *Anesthesiology* 103 821–827. 10.1097/00000542-200510000-00021 16192775

[B38] Oshiro Y. Quevedo A. S. Mchaffie J. G. Kraft R. A. Coghill R. C. (2007). Brain mechanisms supporting spatial discrimination of pain. *J. Neurosci.* 27 3388–3394. 10.1523/jneurosci.5128-06.2007 17392455 PMC6672117

[B39] Oshiro Y. Quevedo A. S. Mchaffie J. G. Kraft R. A. Coghill R. C. (2009). Brain mechanisms supporting discrimination of sensory features of pain: a new model. *J. Neurosci.* 29 14924–14931. 10.1523/jneurosci.5538-08.2009 19940188 PMC2823474

[B40] Raichle M. E. MacLeod A. M. Snyder A. Z. (2001). A default mode of brain function. *Proc. Natl. Acad. Sci. U.S.A.* 98 676–682. 11209064 10.1073/pnas.98.2.676 PMC14647

[B41] Russo A. Marcelli V. Esposito F. Corvino V. Marcuccio L. Giannone A. (2014). Abnormal thalamic function in patients with vestibular migraine. *Neurology* 82 2120–2126. 10.1212/wnl.0000000000000496 24814847

[B42] Sauro K. M. Rose M. S. Becker W. J. Christie S. N. Giammarco R. Mackie G. F. (2010). HIT-6 and MIDAS as measures of headache disability in a headache referral population. *Headache* 50 383–395. 10.1111/j.1526-4610.2009.01544.x 19817883

[B43] Shin J. H. Kim Y. K. Kim H. J. Kim J. S. (2014). Altered brain metabolism in vestibular migraine: comparison of interictal and ictal findings. *Cephalalgia* 34 58–67. 10.1177/0333102413498940 23918837

[B44] Smith A. T. Greenlee M. Deangelis G. C. Angelaki D. E. (2017). Distributed visual–vestibular processing in the cerebral cortex of man and macaque. *Multisens. Res.* 30 1–29. 10.1007/978-1-4757-9628-5_1

[B45] Starr C. J. Sawaki L. Wittenberg G. F. Burdette J. H. Oshiro Y. Quevedo A. S. (2009). Roles of the insular cortex in the modulation of pain: insights from brain lesions. *J. Neurosci.* 29 2684–2694. 10.1523/jneurosci.5173-08.2009 19261863 PMC2748680

[B46] Stephan T. Deutschlander A. Nolte A. Schneider E. Wiesmann M. Brandt T. (2005). Functional MRI of galvanic vestibular stimulation with alternating currents at different frequencies. *Neuroimage* 26 721–732. 10.1016/j.neuroimage.2005.02.049 15955481

[B47] Teggi R. Colombo B. Rocca M. A. Bondi S. Messina R. Comi G. (2016). A review of recent literature on functional MRI and personal experience in two cases of definite vestibular migraine. *Neurol. Sci.* 37 1399–1402. 10.1007/s10072-016-2618-6 27225278

[B48] Tomasi D. Volkow N. D. (2011). Association between functional connectivity hubs and brain networks. *Cereb. Cortex* 21 2003–2013. 10.1093/cercor/bhq268 21282318 PMC3165965

[B49] Ventre-Dominey J. (2014). Vestibular function in the temporal and parietal cortex: distinct velocity and inertial processing pathways. *Front. Integr. Neurosci.* 8:53. 10.3389/fnint.2014.00053 25071481 PMC4082317

[B50] Wang S. Wang H. Zhao D. Liu X. Yan W. Wang M. (2019). Grey matter changes in patients with vestibular migraine. *Clin. Radiol.* 74 898.e1–898.e5. 10.1016/j.crad.2019.07.015 31451181

[B51] Yan C. G. Wang X. D. Zuo X. N. Zang Y. F. (2016). DPABI: data processing & analysis for (resting-state) brain imaging. *Neuroinformatics* 14 339–351. 10.1007/s12021-016-9299-4 27075850

[B52] Yang F. C. Chou K. H. Lee P. L. Yin J. H. Chen S. Y. Kao H. W. (2019). Patterns of gray matter alterations in migraine and restless legs syndrome. *Ann. Clin. Transl. Neurol.* 6 57–67. 30656184 10.1002/acn3.680 PMC6331309

[B53] Yuan K. Qin W. Dong M. Liu J. Sun J. Liu P. (2010). Gray matter deficits and resting-state abnormalities in abstinent heroin-dependent individuals. *Neurosci. Lett.* 482 101–105. 10.1016/j.neulet.2010.07.005 20621162

[B54] Zhang J. Su J. Wang M. Zhao Y. Zhang Q. T. Yao Q. (2017). The sensorimotor network dysfunction in migraineurs without aura: a resting-state fMRI study. *J. Neurol.* 264 654–663. 10.1007/s00415-017-8404-4 28154971

[B55] Zhe X. Gao J. Chen L. Zhang D. Tang M. Yan X. (2020). Altered structure of the vestibular cortex in patients with vestibular migraine. *Brain Behav.* 10:e01572. 10.1002/brb3.1572 PMC7177586 32157823

[B56] zu Eulenburg P. Caspers S. Roski C. Eickhoff S. B. (2012). Meta-analytical definition and functional connectivity of the human vestibular cortex. *Neuroimage* 60 162–169. 10.1016/j.neuroimage.2011.12.032 22209784

