# Supplementary material for: Altered Gray Matter Volume and Functional Connectivity in Patients With Vestibular Migraine
Source: Front Neurosci. 2021 Jul 8;15:683802. doi: 10.3389/fnins.2021.683802 (PMC8297163; doi:10.3389/fnins.2021.683802)
Supplement: Supplementary file 1 [file Data_Sheet_1.pdf]

Supplementary Figure 1

Nonparametric test: a

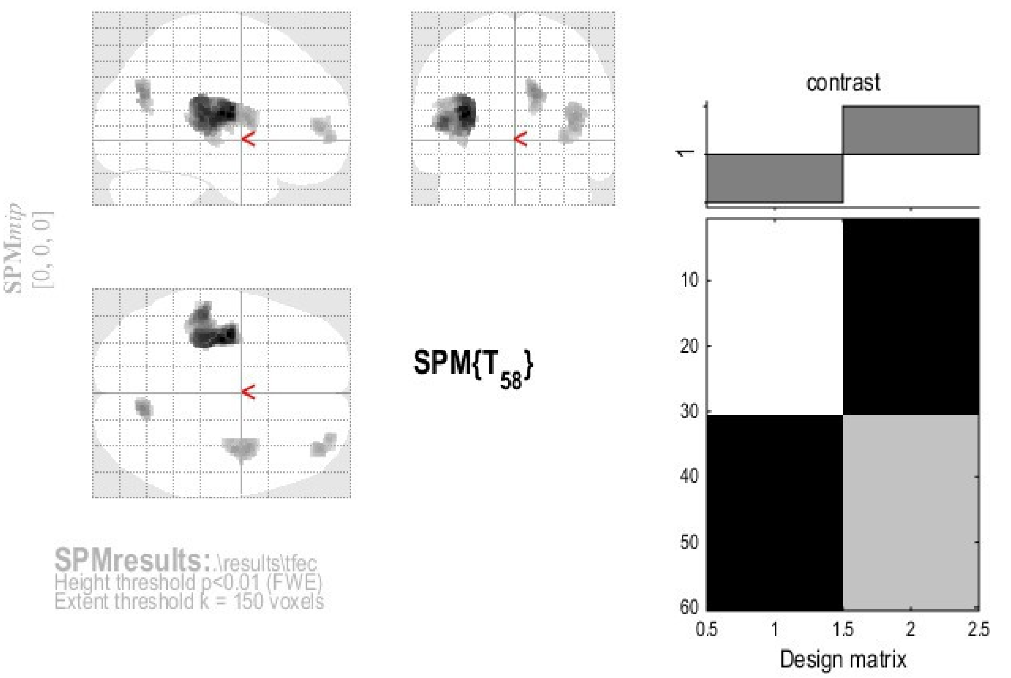

Statistics: ***nonparametric p-values adjusted for search volume***

	peak-level					mm mm mm		
	k_E	$p_{FWE\text{-}corr}$	$q_{FDR\text{-}corr}$	T	p_{uncorr}			
	**1665**	**0.000**	**0.002**	**7.20**	**0.000**	**-36**	**-11**	**14**
		0.000	0.002	6.78	0.000	-32	-20	11
		0.000	0.002	6.74	0.000	-35	-26	15
	**179**	**0.001**	**0.002**	**5.93**	**0.000**	**12**	**-68**	**26**
		0.007	0.002	5.22	0.000	11	-63	17
	**270**	**0.001**	**0.002**	**5.72**	**0.000**	**38**	**-6**	**14**
		0.002	0.002	5.70	0.000	36	3	9
		0.003	0.002	5.51	0.000	39	3	17
	**235**	**0.002**	**0.002**	**5.63**	**0.000**	**38**	**50**	**5**
		0.003	0.002	5.54	0.000	32	57	0
		0.007	0.002	5.22	0.000	36	56	12

table shows 3 local maxima more than 8.0mm apart

Degrees of freedom = [1.0, 58.0]
FWHM = 11.8 12.7 12.7 mm mm mm; 7.9 8.5 8.5 {voxels}
Volume: 1482600 = 439289 voxels = 641.9 resels

Voxel size: 1.5 1.5 1.5 mm mm mm; (resel = 565.84 voxels)
Permutations = 5000

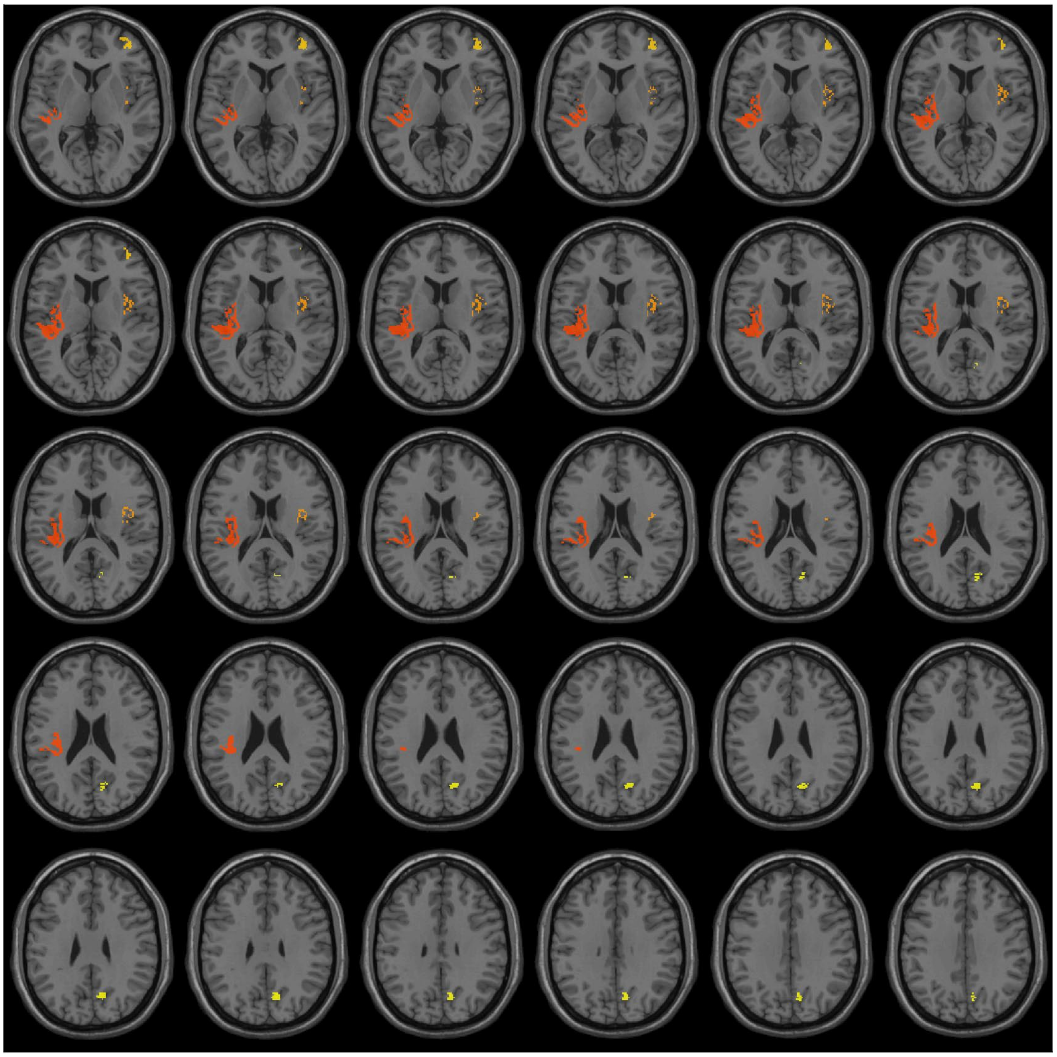

Supplementary Figure 2
